# Gaming Disorder and Well-Being Among Emirati College Women

**DOI:** 10.3389/fpsyt.2021.659508

**Published:** 2021-05-25

**Authors:** Marina Verlinden, Justin Thomas, Mahra Hasan Abdulla Ahamed Almansoori, Shamil Wanigaratne

**Affiliations:** ^1^College of Natural and Health Sciences, Zayed University, Abu Dhabi, United Arab Emirates; ^2^National Rehabilitation Center, Abu Dhabi, United Arab Emirates

**Keywords:** internet gaming disorder, IGD, online gaming, addiction, depression, UAE

## Abstract

**Background:** The present study examined Internet Gaming Disorder (IGD) and depressive symptom levels among a predominantly female sample of college students from the United Arab Emirates (UAE).

**Methods:** IGD was assessed among two successive cohorts of students at the beginning of the academic year in 2016 and 2019, respectively. All participants (*n* = 412) completed the Internet Gaming Disorder Scale – Short-Form (IGDS9-SF) and the WHO-5 Well-being Index (WHO-5), a tool widely used for the screening and assessment of depressive symptomatology.

**Results:** Mean IGDS9-SF scores (15.85, *SD* = 6.40) were fairly similar to those observed in other nations. The prevalence of IGD was 1.45%, based on the stringent cut-off score (> = 40). Prevalence of IGD rose to 18.20% when using the less stringent cut-off (> = 21). There was an increase in the rate of IGD between 2016 and 2019, although not statistically significant. Higher IGDS9-SF scores were associated with greater depressive symptomatology; those scoring above the less stringent IGD cut-off had a greater likelihood of screening positive for depression OR = 2.28, 95% CI (1.176–4.428).

**Conclusions:** This study provides insights about IGD among a predominantly female Arab population, finding a correlation with mood disorder symptomatology and suggesting an increase in problematic gaming over time. The results are discussed with reference to the mood repair hypothesis and the possibility of IGD being a dual disorder. The association with depressive symptoms is also discussed in light of the neurobiology of addictive behaviors and sexual dimorphism.

## Introduction

The World Health Organization's International Classification of Diseases, 11th Revision, (ICD-11) lists gaming disorder in the section on addictive behaviors (1). In the ICD-11, it is described as online or offline persistent or recurrent gaming in individuals, who experience loss of control over gaming, disproportionately prioritize gaming over other important activities, and continue gaming despite the negative impact of gaming on their daily lives (1). In acknowledgment of this growing health threat, the American Psychiatric Association also included Internet Gaming Disorder (IGD) in the fifth edition of the Diagnostic and Statistical Manual of Mental Disorders (DSM-5) (2). In the DSM-5, IGD is described as a repetitive gaming resulting into a significant impact on individuals functioning, marked by some of the following symptoms: preoccupation with gaming, loss of interest in daily activities, a build-up of tolerance, withdrawal symptoms, failed attempts to stop gaming, lying to others about gaming, gaming to escape anxiety or guilt emotions, as well as losing or putting at risk relationships or opportunities due to gaming behaviors (2).

Reported prevalence of IGD ranges from as low as 1.2% (3) to as high as 14.6% (4) depending on a country, age of the sample and the study methodology. Overall, the average prevalence based on studies from the past two decades is estimated to be 4.7% (5). Current research suggests that IGD is more common among younger age groups (6, 7) and among males (7, 8). However, market research suggests that proportion of female gamers is increasing, particularly among mobile phone gamers (9). The existing and rapidly expanding body of research on IGD suggest that the condition is associated with a matrix of physical, emotional and behavioral concerns, including poor sleep quality (3, 6, 10–12), overweight/obesity (13), lower academic achievement (3, 6), aggressive and disruptive behaviors (14), depression and anxiety (14–18), as well as a considerable financial burden due to regular in-app purchases (spending real-world money for in-game assets such as weapons, lives, pets) (19–22). The psychosocial ramifications of excessive gaming are particularly alarming (23). Such concerns about IGD and its potentially negative impact on child and adolescent developmental trajectories underpin the need for a better understanding of this condition.

The American Psychiatric Association called for studies of IGD across different populations and cultures (2). One of the main goals of including the IGD into the DSM-5 Section 3 was to generate cross-cultural data regarding the proposed diagnostic criteria and to determine its prevalence across different populations (24). However, IGD remains relatively understudied among female gamers and among populations from the Middle East and North Africa. At the time of writing we found no systematic studies of IGD among citizens of the relatively wealthy Gulf Arab states (e.g., Kuwait, Saudi Arabia and the United Arab Emirates), apart from a report of a pilot study among university students in the UAE (Wanigaratne et al., submitted)^1^. The few studies from the region do not focus on IGD but describe the broader issue of problematic Internet use and Internet addiction (25–28). The United Arab Emirates (UAE) has one of the highest Internet penetration rates worldwide. According to market research and industry watch dogs, 99% of the country's population are considered active internet users, of which 92.2% are mobile internet users spending on average 7 h online daily (29). Similarly, the UAE is reported to have one of the highest percentages (92%) of smartphone owners among the country's Internet users (30). The same report also suggests that 80% of internet users in the UAE play online games, with 65% using a smart phone to do so (30). The high Internet and mobile penetration rates and the strong connection to Gulf Arab heritage make the UAE a potentially useful context to explore the prevalence and correlates of IGD in this region. To the best of our knowledge, no previous studies up till now have examined the prevalence of IGD in the UAE or broader Arabian Gulf region. Given UAE's high Internet penetration rates and the recent gender demographic trend in mobile gaming, we aimed to examine the prevalence of IGD with a special focus on female gamers to identify the prevalence of DSM-5 endorsed criteria for IGD among Emirati college students studying in the UAE. We undertook this study at two distinct time-points, 2016 and 2019, hypothesizing an increasing prevalence at time two. We also investigated the association between IGD and depressive symptoms, hypothesizing IGD to be associated with elevated depressive symptomatology. Furthermore, we recognize the immense complexity of the neurobiological mechanisms involved in addictions (31, 32). Current research perspectives offer an understanding of the addictions as disorders resulting from a web of interconnected mechanisms. Rather than viewing addiction as being merely an outcome of excessive engagement in one specific behavior, such as gaming, they are viewed as involving a matrix of brain processes, genetics and sexual dimorphism (32). Behavioral addictions, such as gambling disorder or excessive gaming, can also be viewed as dual disorders (32). Similar to substance related disorders, behavioral addictions are likely to occur in tandem with other mental disorders. In our study, we consider the dual perspective on addictions through examining the association between IGD and depression, and we discuss the study results in light of the clinical neuroscience, sexual dimorphism and the mood-repair hypothesis.

## Methods

### Participants and Measures

Participants were recruited via an email announcement sent to all students. Two independent cohorts of college students completed the online assessment, the first cohort completed in September 2016 (*n* = 108), while the second cohort completed the assessment in September 2019 (*n* =304). The language of tuition at the participating institution is English and all students are expected to be highly bilingual in English and Arabic. Participants reported demographic information (i.e., age, gender, marital status and nationality), before going on to completing Internet Gaming Disorder Scale – Short-Form (IGDS9-SF) (33) and the WHO-5 Well-being Index (34). Both instruments were presented in dual language form, with English alongside Arabic.

IGDS9-SF is nine-item measure of gaming disorder where each item reflects one of the APA's DSM-5 proposed criteria for IGD. Respondents are asked to rate the nine statements about their gaming experience, with reference to the preceding 12 months. More specifically, the instructions that are provided to the participants along with this instrument are as following: “These questions will ask you about your gaming activity during the past year (i.e., last 12 months). By gaming activity, we understand any gaming-related activity that has been played either from a computer/laptop or from a gaming console or any other kind of device (e.g., mobile phone, tablet, etc.) both online and/or offline.” Based on responses to a 5-point scale, varying from 1 = “never” to 5 = “very often,” the IGDS9-SF scores can range from 9 to 45, with higher scores indicative of greater IGD symptomatology. IGDS9-SF has been widely used and well-validated (5, 33). Its reliability in the current study was α = 0.86. Pontes and Griffiths (33) indicated that the main purpose of the IGDS9-SF is not to diagnose the IGD but to assess severity of its symptoms, using it as a continuous score where higher scores are considered indicative of a higher degree of Internet Gaming Disorder. In current literature, for research purposes, different cut-offs have been proposed for categorization of IGD severity as disordered gaming (33, 35). Pontes and Griffiths described rather stringent cut-off scores of 36 and 40 (i.e., equivalent to rating all the nine statements as “often” or “very often” occurring) that can be used to identify suspected cases of IGD (33). However, Monacis et al. suggested an a cut-off > = 21 which was identified based on the empirical analyses of the ROC curve (35). In the present study, in order to examine the prevalence of IGD, we apply both cut-offs currently reported in the literature: the most stringent cut-off (> = 40) and the more lenient cut-off (> = 21).

The WHO-5 Well-being Index (WHO-5) is a short self-report of current psychological well-being, which has been effectively used in screening for major depressive disorder (36). The inventory consists of five statements assessing psychological well-being over the preceding 2 weeks. Each item is rated using a 5-point scale, ranging from 0 = “at no time” to 5 = “all of the time.” The total raw score of 0 to 25 is multiplied by 4 to calculate the standardized percentage score ranging from 0 to 100, where 100 represents the best possible well-being. Scores below 50 are viewed as potentially problematic, perhaps indicative of a depressive disorder (34). In the present study, the internal reliability of the WHO-5 was also good, α = 0.89.

### Procedure, Statistical Analysis, and Ethics

The study procedures were carried out in accordance with the Declaration of Helsinki. The Institutional Review Board of Zayed University (Abu Dhabi, UAE) approved the study (ZU20_045_F). All subjects were informed about the study and all provided informed consent. All data were collected online, via a private link sent to participating students. Participants completed demographic data, followed by the IGDS9-SF and finally the WHO-5. Statistical analyses were performed using IBM SPSS Statistics for Macintosh, Version 27. Descriptive analyses included frequencies and measures of central tendency. In the inferential analysis, Pearson's correlation coefficient was calculated to examine the association between IGD scores and depressive symptoms. Logistic regression analysis was used to examine the association between participants' scores for depressive symptoms in relation to dichotomized IGD symptoms (categories were identified based on the above-described cut-offs). The independent groups *T*-test statistic was calculated to examine the differences in IGD prevalence across two time points.

## Results

Of the 412 participants, 88.1% were female, and 90.8% were Emirati (others were citizens of the neighboring Gulf countries). Mean age for participants (*n*= 412) was 21.89 (*SD* = 3.80). There were no age or gender-composition differences between the 2016 and 2019 cohorts. The participating institution is gender-segregated, with separate campuses for males and females, with females making up the bulk of the student body. All participants were citizens of the UAE or neighboring Gulf States. Females comprised 88.1% of the sample, reflecting the institution's historical focus on educating Emirati women. All continuous variables were normally distributed, although IGDS9-SF scores were slightly left skewed. Table 1, below, details the descriptive statistics for the study's key variables broken-down by gender.

**Table 1:** Descriptive statistics by gender for the study's key variables.

	**IGDS9-SF**			**WHO-5**			**Age**		
	**All**	**F**	**M**	**All**	**F**	**M**	**All**	**F**	**M**
Median	14.00	13.00	19.00	64.00	64.00	76.00	22.00	22.00	19.00
IQR	11–19	10–19	16–22	44–76	44–76	68–100	20–23	20–23	18–22
Mean	15.99	15.52	19.49	60.92	60.35	74.66	21.89	22.00	21.06
SD	6.70	6.45	7.486	23.26	23.08	24.73	3.80	3.57	5.18
Min	9.00	9.00	9.00	0.00	0.00	24.00	15.00	15.00	15.00
Max	45.00	45.00	45.00	100.00	100.00	100.00	45.00	45.00	43.00
IGD>=21	18.20%	17.08%	26.53%	–	–	–	–	–	–
IGD>=40	1.45%	1.1%	4.08%	–	–	–	–	–	–

*F, female; M, Male; % IGD > = 21, % screening positive for possible IGD when cut-off employed was > = 21. IGD > = 40, % screening positive for possible IGD when cut-off employed was > = 40*.

The mean age of the participants was 21.9 (*SD* = 3.8) years. There were no gender differences for age. The mean score on the IGDS9-SF was 15.85 (*SD* = 6.40) across the whole sample. Compared to females (*M* = 15.52, *SD* = 6.45), the mean of IGDS9-SF in males was statistically significantly higher 19.49 (*SD* = 7.48), *t* (410) = −3.95, *p* < 0.001. Applying the cut-off score of > **=** 40 to the IGDS9-SF scores yielded the prevalence of IGD 1.5%. After the application of the less stringent cut-off **>** = 21, the prevalence of those with disordered gaming was 18.2%. The item level analysis showed that the highest observed mean score was for the item “*Do you play in order to temporarily escape or relieve a negative mood”* (*M* = 2.51, *SD* = 1.32), showing this IGD criterium was most frequently endorsed by the participants. This IGDS9-SF criterion represents the Escape criterion of IGD in the DSM-5. This IGD item was the only item with the median of 3 (other items medians ranged from 1 to 2). Furthermore, this item was most frequently endorsed (9.70% of respondents, *n* = 40) with the response “strongly agree” (score of 5). The next closest item with a high endorsement rate (i.e., score 5) was IGD's item 6 (“Have you continued your gaming activity despite knowing it was causing problems between you and other people”) with 4.39% of respondents endorsing it with “strongly agree” (a score of 5).

The WHO-5 mean score in our sample was 15.23 (*SD* = 5.81). Our results showed a weak negative correlation between the IGDS9-SF scores and the WHO-5 scores *r* = −0.17, *p* = 0.04. Table 2, above, details the correlations between the study's main variables.

**Table 2:** Pearson's correlation co-efficients for the study's main variables.

	**Gender**	**WHO-5**	**IGDS9-SF**
Age	−0.08	0.19	−0.12^**^
Gender		0.12	0.19
Depression			−0.17^*^

* *p < 0.05*,

** *p < 0.01*.

Further exploring the relationship between depressive symptoms and IGD, using bivariate logistical regression analysis, we observed that participants scoring above the cut-off (> = 21) on the IGDS9-SF were at higher risk of screening positive for depression (WHO-5 scores below 50), OR = 2.281, 95% CI (1.176–4.428), *p* = 0.015. Conducting the same analysis with the more stringent cut-off (> = 40) may preclude meaningful estimations as the group categorized as disordered gamers using this cut-off was very small, and this analysis resulted in a non-significant result, OR = 0.800, 95% CI (0.082–7.834), *p* = 0.8.

Finally, a comparison of the IGDS9-SF scores in 2016 and 2019 showed that the prevalence of suspected IGD increased from 0.9% in 2016 to 1.6% in 2019 (when applying a cut-off score of >= 40). Among female participants the prevalence in 2016 was 0% rising to 1.4% in 2019. Application of the less stringent cut-off score (> = 21) revealed an increase from 15.7 to 19.1% between 2016 and 2019 across the whole sample. Among females the increase went from 12 to 18% (cut-off score > = 21). Although directionally supportive of the hypothesis, independent groups *t*-test revealed that these increases were not statistically significant.

## Discussion

This study, to the best of our knowledge, is one of the few to examine the prevalence of IGD in the Arabian Gulf region. Focusing primarily on Emirati college women, our findings suggest that a small proportion of these students have levels of IGD symptomatology severe enough to meet proposed criteria for gaming disorder. While IGDS9-SF has been widely used in Western and Asian societies, this study extends the international and cross-cultural applicability of the gaming disorder construct to Arab college students in the UAE. The current findings suggest that mean IGDS9-SF scores are roughly equivalent to the scores observed in college student samples across several other nations in similar studies undertaken between 2016 and 2019 (35, 37–39). The similarity in scores further supports the idea that IGD is a global problem, emerging among all communities where electronic games are readily available. The similarity of scores and the correlation with depression also suggest the IGDS9-SF is potentially a useful screening instrument among Emirati college students, either for the purpose of early detection or research. However, more research is needed to establish the empirical and clinical validity of the cut-off scores (33, 35) when using IGDS9-SF.

In line with previous international research (7, 8), the prevalence of IGD in our sample was higher among male participants. An increase in the IGD prevalence across the two time points may be indicative of a future trend. This increase, although not statistically significant, was observed for both genders. However, it was particularly pronounced among females. The rise of the IGD over time may reflect the high internet penetration in the UAE as well as the increased mobile gaming among females globally (9, 40). The growing market of mobile gaming is increasingly driven by female gamers (9) and this may bring about changes in the IGD demography, perhaps resulting in higher levels of IGD symptomology among females in the time to come. It is important also to consider gender roles in the Gulf, that generally place greater restrictions on the freedom of movement for females. Such restrictions can make the engagement in gaming and online gaming among females more appealing. This could be examined in future studies.

As hypothesized, the IGD was correlated with depressive symptoms, which is also in line with the previous research demonstrating this comorbidity (14–18). The direction of the association between gaming and depression, and the exact role of depression in relation to disordered gaming, are still debated. There is some evidence for a comorbidity (16, 17), some for a mediating effect (15), some for a possible reciprocity in the relationship between gaming and depression (16, 41), and some evidence of a prospective effect of gaming on depression (42).

Importantly, the association of IGD with depression, and the observed sex differences, should be examined from the perspective of neurobiology and genetics. This perspective offers important insights about the similarities between IGD and other addictions, comorbidities and sex differences (sexual dimorphism of the brain across psychiatric disorders).

Addiction can be conceived as a complex brain disorder, impacted by genetic, neurobiological, psychological and social factors (32). Behavioral addiction should be understood as a network of interconnected processes in the human brain that affect thinking, perceiving, decision-making and self-control, rather than a specific behavior, such as excessive gaming or gambling (31, 32). Presumably, vulnerabilities to addictions have some shared mechanisms. Behavioral addictions are marked by problems in brain regions such as the frontal cortex and limbic system (43). Some of the neuroadaptations and alterations of plasticity in the brain regions are similar in behavioral addictions and substance addictions (43). Similar brain regions are activated during engagement in behavioral addictions (e.g., video gaming, gambling) as seen in people with substance-related addictions (43). Similar to people with substance addictions, the neurobiological characteristics observed in people experiencing IGD include problems with response-inhibition, emotion regulation, cognitive control, impaired working memory and decision-making, as well as deficits in the neuronal reward system (44). Furthermore, while some differences in biological and psychological markers of IGD exist (45), studies of the neurobiological correlates of IGD suggest that IGD and substance addictions are likely to have some common predisposing factors (44, 46). Current knowledge suggests that underlying neurobiological mechanisms of IGD involve dopamine-mediated reward system, impulse control and decision making problems, as well as problems in brain networks related to cognitive control, executive function, motivation and reward (47). Furthermore, video games that have a high level of novelty and speed appear to trigger the release of endogenous dopamine at similar levels to exogenous dopamine released from cocaine and amphetamines (48). Decreased neuronal dopamine seen in people with IGD has been proposed as one of the possible common underlying mechanisms for a vulnerability to gaming as well as major depressive disorder (23).

Researchers in the field of addictions suggest that for most of the addictions it is a reasonable expectation that they develop as dual disorders (32) – disorders that are likely to occur in tandem with one or more other mental health disorders (e.g., addiction and depression as examined in this study). This notion is derived from the genetic explanation for the commonalities across the phenotypes and endophenotypes (such as, for example, impulsivity and sensation seeking) that are present across different mental disorders (32). These commonalities may reflect a vulnerability to co-occurring mental disorders. For example, similar to substance addictions, IGD appears to occur with other mental health conditions, such as attention deficit hyperactivity disorder (ADHD) (49), sleep problems, dysphoria and depression (31). Such psychiatric comorbidities are also observed in people with disordered gambling, including the comorbidity with ADHD and depression, among others (32). Studies, that focused on this, are suggesting there may be shared genetic and environmental factors which could be contributing to development of such comorbidities in addiction disorders (50). It is hoped that studies adopting the dual perspective to addiction research will help to identify the vulnerability characteristics, distinctive endophenotypes as well as neurobiological, behavioral and cognitive vulnerabilities to addictions and other mental disorders (32). In line with the above ideas, our study also found and association between IGD and depressive symptoms, supporting the idea that IGD may be manifesting as dual disorder among those with shared IGD/depressive vulnerability.

We might also consider the sex differences observed in the present study and the IGD literature in general. Psychiatric disorders are often described in terms of sex differences (difference in men and women with regard to the prevalence of the disorder, its symptoms and differential response to treatment). In this study it was also confirmed that, vulnerability to depression and gaming disorder is different in men and women. Sexual dimorphism of the brain is an important aspect in understanding the association between IGD and depression. This sexual dimorphism, is an expression of the differences in men and women with regard to their genetics, brain circuits, number of receptors, receptor binding, and signaling as well as hormonal differences in men and women (32). This is also observed in the behavioral and emotional responses of men and women. Women, for instance, tend to attribute their engagement in gambling to stressful and depressive states, while men seem to be driven by sensation seeking, risk-taking and impulsivity (32). Similar patterns of sexual dimorphism are likely to occur in the context of IGD. Future studies could explore this in more detail by including the trait of impulsivity, as an endophenotype. Evolutionarily, impulsivity tends to be higher in men, however, it is also present in women, and this endophenotype is strongly associated with the presence of IGD and other addictive disorders. In addition, future studies could explore the role of ADHD, where high impulsivity and attention deficits are pronounced, and explore this dual phenotype (i.e., depression in tandem with IGD) in greater detail. Brain and hormonal differences between men and women are possible explanations for the emotional and behavioral differences between men and women. In our study, we also observed manifestations of such sexual dimorphism: while women are known to have higher vulnerability to depression, gaming was higher in men, as men tend to be higher on impulsivity and sensation-seeking, and yet the association between gaming and depression was present in both men and women. The sexual dimorphism of the brain is likely to influence symptom expression in the relationship between IGD and depression.

Furthermore, some research suggests that people vulnerable to attention problems may engage in continuous internet gaming as means of enhancing their attention ability (49), while those with depressive symptoms may engage in excessive gaming as means of mood repair or experiential avoidance, as a form of “self-medication.” In our study the most frequently endorsed IGD criterion was: gaming in order to temporarily escape or relieve a negative mood. We suggest that there may be a relation to mood and affective problems possibly through a *mood repair* function of gaming. The *mood repair hypothesis* postulates that people experiencing unpleasant mood states divert the attention away from the self. Some excessive gamers may engage in gaming to manage, repair or escape from their unpleasant moods. Such experientially avoidant behaviors can become maladaptive, requiring increasing amounts of time and focus. Other researchers concur, and report that gaming to “escape adverse moods” is one of the most frequently reported IGD criteria (3). While this criterion in itself is less likely to be indicative of the IGD diagnosis, it does support the mood repair hypothesis for the role of gaming in relation to depression. This, again, could be more fully examined in future research.

In sum, the association between gaming and depression observed in our study supports the notion of addictions being dual disorders, and that gaming disorder is likely to occur in tandem with other psychiatric disorders, such as depressive symptoms, as examined in our study. Our findings, and previous similar research discussed above, could be seen as potential evidence that, similar to gambling disorder, IGD may be a dual disorder rather than a single nosological entity (32), and that the severity of the depressive symptoms is likely to be correlated with the severity of gaming disorder symptoms. Finally, the findings suggest that gaming may function as a mood repair mechanism in people with depressive symptoms.

While the present study extended the exploration of IGD to a Gulf Arab population, it also has several significant limitations. The reliance on a self-selecting convenience sample limits the generalizability of the results. Similarly, the collection of data within a single institution also, somewhat, limits the generalizability to the broader female college population. However, as an initial investigation of this important phenomenon within the UAE, the study establishes the need to undertake more robust investigations, perhaps with a prospective longitudinal design to explore the trajectory of gaming behavior across time and evaluate the degree of social and occupational impairment associated with scores above the IGDS9-SF cut-offs. Given the variation in the prevalence found in the two UAE studies, a nationwide study is needed to establish prevalence rates across age and gender, and to inform policies and interventions for prevention and treatment of IGD. Future studies might also explore religio-cultural factors that may constitute risk or resilience factors in the development and maintenance of IGD.

## Data Availability Statement

The datasets presented in this article are not readily available because the ethical approval for the study requires that only anonymized data may be shared on request with verified researchers. Requests to access the datasets should be directed to Justin Thomas, Justin.Thomas@zu.ac.

## Ethics Statement

The studies involving human participants were reviewed and approved by Zayed University Research Ethics Committee. The patients/participants provided their written informed consent to participate in this study.

## Author Contributions

JT contributed to conception of the project, analysis, and drafting of the manuscript. MV contributed to obtaining IRB approval and drafting of the manuscript. MA contributed to the data collection and drafting of the manuscript. SW contributed to drafting of the manuscript. All authors contributed to the article and approved the submitted version.

## Conflict of Interest

The authors declare that the research was conducted in the absence of any commercial or financial relationships that could be construed as a potential conflict of interest.

## Footnotes

^1^Wanigaratne S, Fikri A, Ahmad AS, Al Marzooqi A, Elsori D, Khalil A, et al. Investigating the prevalence of internet gaming disorder (IGD) in a university student population in the United Arab Emirates: a pilot study. (2020).
